# RGS2 promotes estradiol biosynthesis by trophoblasts during human pregnancy

**DOI:** 10.1038/s12276-023-00927-z

**Published:** 2023-01-18

**Authors:** Chao Tang, Meiyuan Jin, Bingbing Ma, Bin Cao, Chao Lin, Shouying Xu, Jiayong Li, Qiang Xu

**Affiliations:** 1 National Clinical Research Center for Child Health of the Children’s Hospital, Zhejiang University School of Medicine, Hangzhou, 310052 China; 2 Department of Obstetrics, Tongde Hospital of Zhejiang Province, Hangzhou, 310012 China; 3 Institute of Pharmaceutical Biotechnology & Research Center for Clinical Pharmacy, The First Affiliated Hospital, School of Medicine, Zhejiang University, Hangzhou, 310058 China; 4 Zhejiang Provincial Center for Disease Control and Prevention, Hangzhou, 310057 China; 5 Department of Neurosurgery, The Children’s Hospital, Zhejiang University School of Medicine, Hangzhou, 310052 China; 6 State Key Laboratory of Ophthalmology, Zhongshan Ophthalmic Center, Sun Yat-sen University, Guangzhou, 510060 China

**Keywords:** Developmental biology, Endocrine reproductive disorders

## Abstract

Production of estradiol (E2) by the placenta during human pregnancy ensures successful maintenance of placental development and fetal growth by stimulating trophoblast proliferation and the differentiation of cytotrophoblasts into syncytiotrophoblasts. Decreased levels of E2 are closely associated with obstetrical diseases such as preeclampsia (PE) in the clinic. However, the mechanisms underlying the inhibition of placental E2 biosynthesis remain poorly understood. Here, we report that regulator of G-protein signaling 2 (RGS2) affects E2 levels by regulating aromatase, a rate-limiting enzyme for E2 biosynthesis, by using human trophoblast-derived JEG-3 cells and human placental villus tissues. RGS2 enhanced the protein degradation of the transcription factor heart and neural crest derivatives expressed 1 (HAND1) by suppressing ubiquitin-specific protease 14 (USP14)-mediated deubiquitination of HAND1, resulting in the restoration of HAND1-induced trans-inactivation of the *aromatase* gene and subsequent increases in E2 levels. However, aromatase bound to RGS2 and repressed RGS2 GTPase activating protein (GAP) activity. Moreover, we observed a positive correlation between RGS2 and aromatase expression in clinical normal and preeclamptic placental tissues. Our results uncover a hitherto uncharacterized role of the RGS2-aromatase axis in the regulation of E2 production by human placental trophoblasts, which may pinpoint the molecular pathogenesis and highlight potential biomarkers for related obstetrical diseases.

## Reproduction: a placental pathway to pre-eclampsia

Pre-eclampsia can arise from mutations that affect the production of estradiol, a hormone with a critical role in sustaining the placenta during fetal development. Roughly one in 20 pregnancies are affected by this complication, which can put both mother and child at risk and typically manifests as high blood pressure and elevated levels of protein in the urine. The causes of pre-eclampsia are poorly understood, but Chao Tang of Zhejiang University School of Medicine, Hangzhou, China, and colleagues have now linked this condition to defective hormone synthesis. Working with cultured human cells and mouse models, they showed that mutations in a gene called *RGS2* disrupt expression of an enzyme that produces estradiol in the placenta. The researchers also determined that RGS2 expression is consistently lower in placentas from mothers with pre-eclampsia than from unaffected mothers.

## Introduction

As a unique organ of the feto-maternal unit during pregnancy, the human placenta not only physically anchors the fetus to the maternal uterus and coordinates the “crosstalk” between the embryo and the maternal body but also plays an important role as an endocrine unit for the production of hormones to maintain placental development and ensure successful fetal growth^1,2^. Unlike other species, in humans, the functions of the ovary gradually decline after fertilization, while the placenta concurrently develops into the primary site of estrogen biosynthesis. As a result, by 7 weeks of gestation, almost all estrogens in the circulation of the maternal body are produced by the placenta^3^.

The biosynthesis of estrogens in the human placenta is conducted by trophoblast cells^4^. In the human placenta, androgens derived from the maternal and fetal adrenal glands are converted into 17β-estradiol (E2, the active form of estrogens) by the enzymatic action of placental aromatase, an enzyme encoded by the *CYP19a* gene, among which androstenedione is converted into estrone (E1), while testosterone is converted into E2^5–7^. Previous studies have found that the circulating levels of testosterone and androstenedione are increased in pregnant women with preeclampsia (PE), one of the hypertensive syndromes that occurs during pregnancy, compared to normal pregnancies, whereas E2 is decreased in PE^8,9^. This finding suggests that alterations in placental steroidogenesis, such as E2 production and, subsequently, in the functionality or bioavailability of placental aromatase, are mechanistically involved in the pathophysiology of obstetrical diseases, such as PE.

PE, affecting an estimated 4–5% of pregnancies worldwide and resulting in a substantial burden of maternal and fetal morbidity and mortality, is defined as the presence of new-onset hypertension and proteinuria or other end-organ damage occurring after 20 weeks gestation, with risk factors including young maternal age, previous preeclampsia, twin pregnancy, chronic hypertension, diabetes mellitus, and hydatidiform mole^10–14^. Despite breakthroughs in the understanding of PE etiopathogenesis, the physiopathology that triggers the disease is still not completely understood. Given that the development of PE during pregnancy requires the presence of the placenta, it is currently widely accepted that premature placental delivery is the most effective way to terminate this clinical syndrome^15^.

Some genes are associated with human pregnancy maintenance, among which regulator of G protein signaling-2 (RGS2) is recognized as a risk factor for the development of obstetrical diseases such as PE^16^. A specific single nucleotide polymorphism (SNP) in the 3' untranslated region of the *RGS2* gene (rs4606) in mothers is associated with PE^17–19^, and either mutation of *RGS2* or downregulation of RGS2 is associated with hypertension^20–22^. Moreover, knockout of *Rgs2* (*Rgs2*^*−/*−^) was shown to increase resistance but reduce flow in uterine arteries of nonpregnant mice, and disruption of *Rgs2* is sufficient was initiate selected characteristics of PE in pregnant C57BL/6 J mice^16,23^, suggesting that RGS2-regulated downstream signaling contributes to PE. Thus, exploration of the mechanisms underlying RGS2-mediated biological events during pregnancy would provide a better understanding of PE occurrence and progression.

Here, we demonstrate that the decreased levels of RGS2 contribute to the downregulated E2 production during PE progression through the upregulated expression of HAND1 *via* USP14-mediated deubiquitination, which suppresses the transcription of the rate-limiting enzyme aromatase in E2 biosynthesis within human placenta. Our results thereby provide a clue regarding how RGS2 helps maintain human pregnancy and may pinpoint potential therapeutic strategies for related obstetrical diseases.

## Materials and methods

### Cell culture

The human trophoblast-derived cell line JEG-3 and the human embryonic kidney 293 T (HEK293T) fibroblast cell line were obtained from the ATCC. JEG-3 cells were cultured in RPMI1640 medium (Invitrogen, Carlsbad, CA, USA) with 10% fetal bovine serum (FBS), 100 units/ml penicillin and 100 mg/ml streptomycin in 5% CO_2_ at 37 °C, and cells were passaged at a ratio of 1:3 when they were ~90–100% confluent. HEK293T cells were maintained in DMEM complete medium (Invitrogen) with 10% FBS as described previously^5^.

### Plasmids, viruses and infections

RGS2-expressing and aromatase plasmids were amplified by PCR in the presence of cDNA from JEG-3 cells, and the PCR product was subcloned into the eukaryotic expression vector pCDNA3.0 (Invitrogen). The RGS2-expressing lentiviral plasmid was constructed by subcloning human RGS2 into pCDH-CMV-MCS-EF1-copGFP. All of these constructs were verified by a DNA sequencer. Lentiviruses were generated by cotransfecting HEK293T packaging cells with lentiviral expression vector, and lentivirus-containing supernatants with titers >1 × 10^6^ cfu/ml were used for infection of JEG-3 cells or cultured human primary cytotrophoblasts in the presence of 8 μg/ml polybrene (Sigma, St. Louis, MO, USA).

### Quantitative real-time PCR (qRT‒PCR)

Total RNA from JEG-3 cells was isolated with TRIzol reagent (TaKaRa Biotechnology, Dalian, China) following the instruction manual. The complementary DNA (cDNA) was converted with an RNA template using the reverse transcriptase reaction performed according to the manufacturer’s protocol (TaKaRa Biotechnology, Dalian, China). After the termination of cDNA synthesis, the mRNA levels of target genes were determined by quantitative RT‒PCR (qRT‒PCR) as described previously^24^. The relative mRNA levels of the target genes were normalized to that of *α-Tubulin*, and the relative difference in mRNA levels was calculated by the 2^−ΔΔCt^ method.

### Western blot

Western blotting was performed as described previously^25,26^. Briefly, JEG-3 cells and human placental tissues were harvested and lysed in RIPA lysis buffer (Beyotime, Shanghai, China) containing protease inhibitor (Beyotime, Shanghai, China) at 48 h after transfection. After centrifugation to clarify the lysate, the proteins were quantified using the Bicinchoninic Acid (BCA) Protein Assay Kit (Beyotime, Shanghai, China) as per the instruction manual. The cell lysates were mixed with one-fifth volume of 5× sodium dodecyl sulfate (SDS) sample buffer (Epizyme, Shanghai, China) and were denatured by heating at 100 °C for 5 min. After centrifugation for clarification, the samples were visualized by 10% SDS-polyacrylamide gel electrophoresis to fractionate the proteins based on their molecular mass. The separated proteins were transferred to polyvinylidene fluoride (PVDF) membranes by wet electrophoretic transfer (Bio-Rad, Hercules, CA, USA), and the PVDF membranes were then incubated with 5% nonfat skim milk in TBS/Tween-20 (TBST) for 1 h to prevent nonspecific antibody binding. Next, the PVDF membranes were incubated overnight at 4 °C with appropriate primary antibodies, including anti-aromatase antibody at a dilution of 1:1000 (Santa Cruz, CA), anti-STS antibody at a dilution of 1:1000 (Santa Cruz, CA), anti-17β-HSD1 antibody at a dilution of 1:1000 (Santa Cruz, CA), anti-17β-HSD2 antibody at a dilution of 1:1000 (Santa Cruz, CA), anti-3β-HSD1 antibody at a dilution of 1:1000 (HuaBio, Hangzhou, China), anti-RGS2 antibody at a dilution of 1:1000 (Santa Cruz, CA), anti-HAND1 antibody at a dilution of 1:1000 (Bioss, Beijing, China), anti-USP14 antibody at a dilution of 1:1000 (HuaBio, Hangzhou, China), anti-ubiquitin antibody at a dilution of 1:1000 (Santa Cruz, CA), anti-Flag antibody at a dilution of 1:1000 (MBL, Beijing, China), anti-Myc antibody at a dilution of 1:1000 (Santa Cruz, CA), anti-GAPDH antibody at a dilution of 1:2000 (Santa Cruz, CA) and anti-α-Tubulin antibody at a dilution of 1:5000 (HuaBio, Hangzhou, China) under gentle agitation. The next day, the membranes were washed in TBST for 5 min under gentle agitation in triplicate and were further incubated with the appropriate anti-rabbit or anti-mouse HRP-conjugated secondary antibodies at a dilution of 1:10000 (Beyotime, Shanghai, China) at room temperature for 90 min. The specific protein bands on the PVDF membranes were visualized with a chemiluminescence kit (Beyotime, Shanghai, China), and the chemiluminescent signals were quantified with an imaging system (Bio-Rad).

### Immunoprecipitation

Immunoprecipitation was applied as described previously^27^. JEG-3 cells were harvested and lysed in lysis buffer containing 100 mM NaCl, 50 mM Tris-HCl (pH 8.0), 5 mM EDTA, 1% Brij35, 2 mM Na_3_VO_4_, 10 mM NaF, 2 mM β-glycerophosphate and 2 mM PMSF and were incubated with the antibody against aromatase or with rabbit IgG and protein A/G plus agarose (sc-2003, Santa Cruz, CA). The beads were then gently washed five times with lysis buffer, and the immune complex was eluted with Western blot sample buffer. Lysates and immunoprecipitates were subjected to western blotting as described above.

### Enzyme immunometric assays (EIAs)

EIAs for E2 were carried out in serum-free culture supernatants harvested from JEG-3 cells or cultured human placental tissues that were incubated with the substrate (testosterone) of steroidogenic enzymes for 4 h as described previously^27^ and in human and mouse blood samples following the instruction manual.

### Collection of human placental tissue and isolation of human cytotrophoblasts

Placental tissues and blood samples were obtained from 15 women with normal pregnancies and 15 women with PE at the Department of Obstetrics, Tongde Hospital of Zhejiang Province. The samples were obtained between 29 and 40 weeks of gestation from Jan to Dec 2020. Patients with PE were defined as having systolic and diastolic blood pressure > 140 and 90 mmHg, respectively, measured at least 6 h apart plus proteinuria ≥ 300 mg/24 h or > 1+ by dipstick test. To obtain similar clinical conditions to PE, we selected a control group with similar gestational ages. The primary human cytotrophoblasts were purified by using a 5-65% Percoll (Sigma, St. Louis, MO) gradient at step increments of 5% and cultured in high glucose DMEM (Invitrogen, Carlsbad, CA, USA) with 10% (v/v) FBS (Life Technologies, Inc., Grand Island, NY) at 37 °C with 5% CO_2_ as described previously^26^. All experimental protocols were approved by the Ethics Committee of Tongde Hospital of Zhejiang Province, and all studies were performed in accordance with the ethical guidelines of the Ethics Committee of the Tongde Hospital of Zhejiang Province. Informed consent was obtained from all patients.

### Immunofluorescence staining

Immunofluorescence staining was performed on chamber slides (Nalge Nunc International, Naperville, IL). JEG-3 cells or human placental tissue slides were fixed in ice-cold 4% paraformaldehyde (PFA) and permeabilized with 0.1% Triton X-100 in PBS (PBST). After incubation with blocking buffer (1% bovine serum albumin, BSA), samples were incubated with primary antibodies against aromatase and RGS2 and were subsequently incubated with Alexa 546-conjugated and Alexa 488-conjugated secondary antibodies (Life Technologies), respectively. Nuclei were counterstained with 4',6-diamidino-2-phenylindole (DAPI). Slides were analyzed by a laser scanning microscope (Zeiss).

### In situ proximity ligation assay

An in situ proximity ligation assay (PLA) was performed according to the manufacturer’s instructions (Olink Bioscience, Uppsala, Sweden). Briefly, human placental tissues were stained with anti-aromatase rabbit purified polyclonal antibody diluted 1:100 and anti-RGS2 mouse monoclonal antibody diluted 1:50. Signals were detected by Duolink® 100 Detection Kit 613 (red), and nuclei were counterstained with DAPI (blue). Each red dot (if any) represents the detection of a protein‒protein interaction complex. The images were analyzed using optimized freeware (BlobFinder) downloaded from The Centre for Image Analysis at Uppsala University.

### Dual-luciferase assays

The promoter region of *aromatase* (nt -2487/+7) was amplified by PCR in the presence of genomic DNA from JEG-3 cells, and the PCR product was subcloned into the pGL3.0-Basic-luciferase vector (Promega) to generate luciferase reporter constructs as described previously^24^. The constructed dual-luciferase vector was cotransfected with Flag-RGS2 (RGS2), RGS2 siRNA or the corresponding control (Con or scrambled) and Renilla into JEG-3 cells. The cells were harvested and lysed 24 h later, and the luciferase activity was measured by the Dual-Luciferase Assay System (Promega) according to the manufacturer’s instructions.

### Chromatin immunoprecipitation assay

Chromatin immunoprecipitation (ChIP) assays were performed by using a commercial kit (Millipore, Billerica, MA) and a method modified from the manufacturer’s protocol as described previously^27^. Briefly, shearing of chromatin DNA was conducted by sonication to produce ~300–400 bp of input DNA, which was then subjected to immunoprecipitation with an anti-HAND1 antibody, anti-RGS2 antibody or control IgG. After the immunoprecipitates were incubated with protein A agarose/salmon sperm DNA, the antibody-protein‒DNA-agarose complex was washed gently and harvested for subsequent reverse crosslinking. The sheared DNA fragments from reverse crosslinking were extracted with a DNA extraction kit for further PCR amplification.

### Preparation of recombinant proteins

Purification of the RGS2: BL21 (DE3) *E. coli* strain and aromatase: BL21 (DE3) *E. coli* strain transformed by the pet28b-GST-RGS2 and pet28b-GST-aromatase plasmids were cultured at 37 °C in LB medium with ampicillin. When the OD600 reached 0.8, the recombinant proteins were inducibly expressed at 18 °C with 100 mM IPTG for 13 h. The obtained *E. coli* was then suspended in Ni-1 buffer with 20 mM Tris-HCl (pH 8.0), 100 mM MgCl_2_, 10 mM imidazole, and 0.3 mg/ml benzamidine, and the soluble fraction that was prepared through ultrasonication (Astrason Ultrasonic Processor XL2020) was incubated with Ni-NTA agarose beads (Qiagen) at 4 °C for 1 h. After the samples were washed with Ni-2 buffer containing 20 mM Tris-HCl (pH 8.0), 100 mM MgCl_2_, and 20 mM imidazole, the recombinant proteins were eluted into Ni-3 buffer with 20 mM Tris-HCl (pH 8.0), 100 mM MgCl_2_, and 250 mM imidazole by Ni-affinity column chromatography. The lysate obtained was incubated with Glutathione Sepharose 4B beads (GE Healthcare) for 1 h at 4 °C and was then applied to GST-affinity column chromatography for washing by W1 buffer with 25 mM Tris-HCl (pH 7.5), 150 mM NaCl, 10 mM β-mercaptoethanol and 1% Triton X-100, and W2 buffer with 50 mM Tris-HCl (pH 8.0), 150 mM NaCl and 10 mM β-mercaptoethanol. The recombinant proteins were finally eluted into W2 buffer with 10 mM glutathione and concentrated *via* ultrafiltration using Amicon Ultra (Millipore).

### GTPase assay

The GTPase assays were carried out using an ATPase/GTPase activity assay kit (Sigma-Aldrich) as per the manufacturer’s protocol as described previously^28^. Briefly, 3 μg of recombinant RGS2 protein in combination with/without 3 μg of aromatase protein was incubated with increasing amounts of GTP in a volume of 30 μl of the reaction buffer provided by the kit at 37 °C for 3 h. The reactions were then terminated by adding 200 μl of the kit reagent and incubating for an additional 30 min at room temperature. The formation of hydrolyzed free phosphate was measured at a wavelength of 620 nm using a spectrophotometer (Thermo Scientific), and the absorbance was compared with a standard curve. The readings of the background blank and negative control reactions were subtracted from the sample readings. The measurements obtained were processed in Microsoft Excel, and then, the data were transferred to GraphPad Prism for Michaelis–Menten kinetics and statistical analysis.

### Wound healing assay

Wound healing assays were performed as previously described^29^. Briefly, 4 × 10^5^ JEG-3 cells were cultured in six-well plates and transfected with the indicated plasmids. After 24 h, the cells were subjected to serum starvation for 12 h. Then, the cells were rinsed with medium to remove unattached cells, and the confluent layer of cells was scratched with a sterile tip to create an artificial wound. Cell migration to the wounded gap was then monitored by microscopy after 12 h, and the distance between the edges of the wound was analyzed using ImageJ software.

### Matrigel invasion assay

Invasion of JEG-3 cells was examined using Matrigel (BD Biosciences, NJ)-coated Transwell inserts (6.5 μm, Costar, Cambridge) containing polycarbonate filters with 8 μm pores as described previously^29^. Briefly, 50 µl of a mixture of Matrigel and medium at a proportion of 1:2 was enclosed by each Transwell membrane. After transfection and culture for 24 h, JEG-3 cells (2 × 10^5^) in 200 μl of serum-free medium were plated in the upper chamber, while 600 μl of medium containing 10% FBS was added to the lower well. After incubation for 12 h, noninvading cells that remained on the upper surface of the filter were removed, and the cells that had passed through the filter and attached to the bottom of the membrane were fixed in methanol, followed by 0.2% crystal violet staining. The numbers of invasive cells in six randomly selected fields from triplicate chambers were counted in each experiment. The migrated cells were counted under a light microscope.

### *Rgs2* gene transgenic mice

Two heterozygous *C57BL/6N-Rgs2* mice (one male and one female, *Rgs2*^*+/*−^) were obtained from Cyagen Biosciences (Suzhou, China) and were mated to generate heterozygous (*Rgs2*^*+/−*^) and homozygous (*Rgs2*^*−/−*^) offspring. For genotyping, tail clips were taken from weanlings (3 weeks old) or fetuses on embryonic day 13.5 (E13.5) and placed in 200 ml of lysis buffer (50 mM KCl, 10 mM Tris-HCl pH 8.3, 2 mM MgCl_2_, 0.1 mg/ml gelatin, 0.45% NP-40, 0.45% Tween 20) containing 0.4 mg/ml proteinase K, and incubated at 55 °C overnight and then at 95 °C for 10 min. PCR Master Mix (2x) (Yeasen, Shanghai, China) and primers were mixed with 400 ng gDNA, and PCR was amplified under the following conditions: 94 °C for 3 min and 35 cycles of 94 °C for 30 s, 60 °C for 35 s, and 72 °C for 35 s. At E13.5, recipient females were sacrificed, fetuses were prepared for extraction of mouse embryonic fibroblasts (MEFs) and each placenta was cut in half. One half was fixed in 4% PFA for morphological analysis by hematoxylin and eosin (H&E) staining, and the other half was stored at −80 °C until further use. All animal care and handling procedures were approved by the Institutional Animal Care and Use Committee of Zhejiang University.

### Ovariectomy, JEG-3 xenografts and treatments

Female Balb/c-nu/nu mice (6 weeks old) were ovariectomized (OVX) and rested for 1 week, and a tumor-bearing mouse model was established by subcutaneous inoculation with xenografts of human JEG-3 cells (5 × 10^6^ cells/site) infected with control lentiviruses, RGS2-expressing lentiviruses or RGS2-expressing lentiviruses together with HAND1-expressing lentiviruses into both the left and right armpits. At Day 14 after inoculation, the mice were sacrificed, and the tumors and uterus were harvested for further analysis, including determination of serum murine and human E2 levels by EIAs, examination of uterine response to E2 by H&E staining of paraffin-embedded uterine sections, measurements of tumor weights and volumes and examination of the proliferating tumor cells by Ki-67 staining. All animal care and handling procedures were approved by the Institutional Animal Care and Use Committee of Zhejiang University.

### Immunohistochemistry staining

Immunohistochemistry staining was carried out by using the Histostain-Plus Kit (Kangwei Reagents, Beijing, China) according to the manufacturer’s instructions as described previously^29^. Briefly, paraffin-embedded placental sections or tumor sections with a thickness of 4 μm were deparaffinized and rehydrated in xylene and subsequently a graded series of ethanol. After antigen retrieval in 10 mM sodium citrate and 10 mM citric acid, tissue sections were treated with 3% H_2_O_2_-methanol solution to quench endogenous peroxidase followed by sequential incubation with normal goat serum for 30 min at room temperature, with a control rabbit or mouse IgG or a primary antibody against aromatase (sc-374176, Santa Cruz, CA), RGS2 (sc-100761, Santa Cruz, CA) or Ki-67 (bs-23103R, Bioss, China) at 4 °C overnight, and with HRP-labeled secondary antibody (Life Technologies) for 30 min. Diaminobenzidine (DAB) solution was used for color development, and the sections were counterstained with hematoxylin.

### Statistical analysis

All the data are expressed as the mean ± S.D. and analyzed by one-way analysis of variance and Tukey‒Kramer multiple comparison tests (SPSS 13.0 J software, SPSS, Inc., Chicago, IL). Statistical significance was defined at *p* < 0.05 and *p* < 0.01. Correlation analyses were analyzed with a Pearson analysis. Experiments were performed independently in triplicate, and representative experiments are shown.

## Results

### Regulation of E2 production by RGS2 through aromatase

**Fig. 1:**
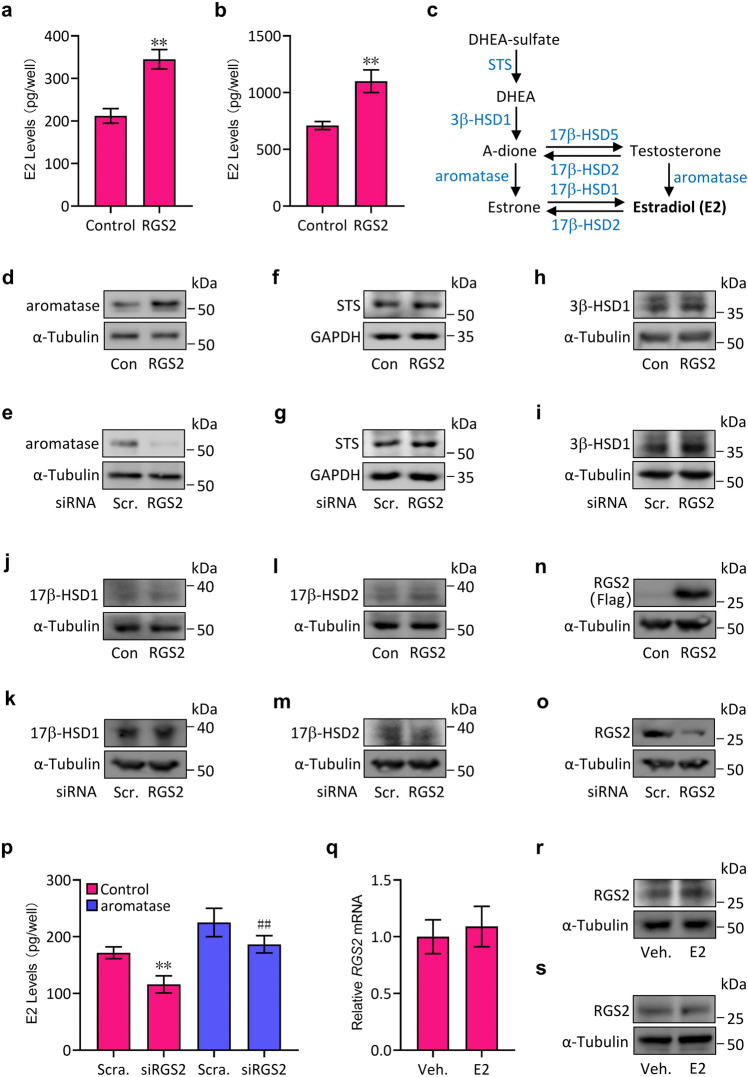
RGS2 regulates E2 levels through aromatase. **a** E2 levels in the culture media of JEG-3 cells transfected with Flag-RGS2 (RGS2) or Flag-tagged empty vector (Con). **b** E2 levels in the culture media of human placental villus tissues after infection with lentiviruses expressing RGS2 or with control lentiviruses for 1 week. **c** A schematic diagram showing the pathway of E2 biosynthesis from DHEA sulfate in human placental trophoblasts. **d**–**o** Western blot analysis for aromatase (**d**, **e**), STS (**f**, **g**), 3β-HSD1 (**h**, **i**), 17β-HSD1 (**j**, **k**), 17β-HSD2 (**l**, **m**), Flag (**n**) and RGS2 (**o**) in JEG-3 cells transfected with Flag-RGS2 (RGS2, **d**, **f**, **h**, **j**, **l**, **n**) or RGS2 siRNA (**e**, **g**, **i**, **k**, **m**, **o**) and the corresponding controls (Con or siRNA Scr.). **p** E2 levels in the culture media of JEG-3 cells transfected with RGS2 siRNA (siRGS2) or scrambled siRNA (Scra.) in combination with aromatase-expressing plasmid or control vector (control). **q** qRT‒PCR analysis of *RGS2* mRNA levels in JEG-3 cells treated with 17β-estradiol (E2) or vehicle (Veh.). **r** Western blot analysis of RGS2 in JEG-3 cells treated with 17β-estradiol (E2) or vehicle (Veh.). **s** Western blot analysis of RGS2 in human primary cytotrophoblasts treated with 17β-estradiol (E2) or vehicle (Veh.). RNA and protein abundance normalized to α-Tubulin or GAPDH, respectively. **p < 0.01 versus Con, scrambled (Scra.) or scrambled (Scra.)+control, ^##^*p* < 0.01 versus RGS2 siRNA (siRGS2)+control; *n* = 6; error bar, SD.

To first test whether RGS2 expression is associated with E2 production, we transfected JEG-3 cells with a Flag-tagged human RGS2-expressing plasmid (Flag-hRGS2, RGS2) or small interfering RNAs (siRNAs) specifically targeting human *RGS2* (hRGS2 siRNA, siRGS2). Our results revealed that overexpression of RGS2 significantly promoted E2 levels by 1.75-fold, and similar results were obtained by using human placental villus tissues infected with RGS2-expressing lentiviruses (Fig. 1a, b), indicating that RGS2 expression affects E2 levels.

Given that E2 synthesis from androgen (such as dehydroepiandrosterone-sulfate, DHEA-sulfate) in human placenta during pregnancy requires steroidogenic enzymes, such as steroid sulfates (STS), 3β-hydroxysteroid dehydrogenase type 1 (3β-HSD1), 17β-hydroxysteroid dehydrogenase type 1 (17β-HSD1) and 17β-hydroxysteroid dehydrogenase type 2 (17β-HSD2) as well as aromatase^5^ (Fig. 1c), we next assessed the protein expression of these enzymes upon exposure to ectopic expression of RGS2 or silencing of RGS2. Consistent with the changes in E2 levels, overexpression of RGS2 markedly increased the protein levels of aromatase, while knockdown of RGS2 resulted in decreased aromatase protein expression (Fig. 1d, e), and the alterations in aromatase expression were further verified by using human placental villus tissues (data not shown). However, the protein levels of other enzymes, including STS, 3β-HSD1 and 17β-HSD1, were not affected by RGS2 (Fig. 1f–k), although the 17β-HSD2 levels revealed slight alterations to some extent (Fig. 1l, m). Moreover, the transfection efficiency of Flag-hRGS2 in JEG-3 cells was verified by the examination of Flag using an anti-Flag antibody, and the knockdown efficiency of RGS2 siRNA (siRGS2) was determined by detecting endogenous RGS2 protein expression (Fig. 1n, o). Consistently, overexpression of aromatase effectively restored the siRGS2-suppressed E2 levels (Fig. 1p), whereas neither RGS2 mRNA expression nor RGS2 protein expression was unexpectedly affected by 17β-estradiol (E2) treatment (Fig. 1q, r), and the unchanged RGS2 protein levels could be further verified in human cytotrophoblasts cocultured with E2 (Fig. 1s), indicating that E2 did not regulate RGS2 expression. Thus, RGS2 modulates the production of E2 likely through the regulation of aromatase expression.

### Enhancement of *Aromatase* transcription by RGS2

**Fig. 2:**
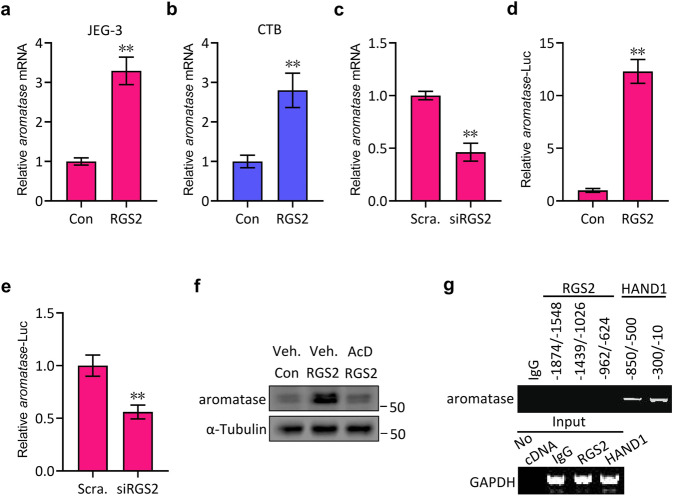
RGS2 regulates *aromatase* transcription. **a** qRT‒PCR analysis of aromatase mRNA levels in JEG-3 cells transfected with Flag-RGS2 (RGS2) or control vector (Con). **b** qRT‒PCR analysis of aromatase mRNA levels in human cytotrophoblasts infected with RGS2-expressing lentiviruses (RGS2) or control lentiviruses (Con). **c** qRT‒PCR analysis of aromatase mRNA levels in JEG-3 cells transfected with RGS2 siRNA (siRGS2) or control siRNA (Scram.). **d** Dual-luciferase assays for the promoter construct of *aromatase* (*aromatase*-Luc) in JEG-3 cells transfected with Flag-RGS2 (RGS2) or control vector (Con). **e** Dual-luciferase assays for the promoter construct of *aromatase* (*aromatase*-Luc) in JEG-3 cells transfected with RGS2 siRNA (siRGS2) or control siRNA (Scram.). **f** Western blot analysis of aromatase in JEG-3 cells transfected with Flag-RGS2 (RGS2) or control vector (Con) and treated with actinomycin D (AcD) or vehicle (Veh.). **g** Detection of the interaction between RGS2 or HAND1 and DNA of *aromatase* promoter constructs by ChIP-PCR assays in JEG-3 cells. RNA and protein abundance normalized to that of α-Tubulin. **p < 0.01 versus Con or siRNA-Scram., respectively; *n* = 6; error bar, SD.

To further investigate the possible regulation of aromatase protein expression by RGS2, we then examined the effect of RGS2 expression on *aromatase* mRNA levels. In accordance with the protein expression data (Fig. 1d, e), overexpression of RGS2 by transfection or lentivirus-mediated infection significantly upregulated *aromatase* mRNA expression in JEG-3 cells and in cultured human cytotrophoblasts (CTBs), respectively (Fig. 2a, b), while inhibition of RGS2 by transfection of RGS2 siRNA effectively downregulated *aromatase* mRNA expression in JEG-3 cells (Fig. 2c), suggesting that RGS2 might affect *aromatase* transcription and that the altered protein levels of aromatase could be derived from changes in its mRNA levels. To gain insight into the potential direct regulation of *aromatase* transcription by RGS2, we cloned the promoter region of *aromatase*, which was subsequently inserted into the pGL3.0-Basic-luciferase reporter vector (*aromatase*-Luc), and performed *aromatase*-Luc reporter assay experiments. As expected, overexpression of RGS2 or knockdown of RGS2 resulted in an obvious increase or apparent decrease in *aromatase*-Luc reporter activity, respectively (Fig. 2d, e), and RGS2-induced aromatase protein expression was obviously attenuated by the presence of actinomycin D (AcD), a specific inhibitor of RNA transcription (Fig. 2f).

Next, we performed chromatin immunoprecipitation followed by PCR (ChIP-PCR) in JEG-3 cells by using an antibody against RGS2 to further examine the potential physical interaction between RGS2 and the promoter region of *aromatase*. As heart and neural crest derivatives expressed 1 (HAND1, a basic helix-loop-helix transcription factor) has been proven to be associated with the *aromatase* promoter^27^, we utilized HAND1 as the positive control in this experiment. The PCR primers were designed to amplify the specific genomic DNA fragments of nt -1874/-1548, nt -1439/-1026 and nt -962/-624 that contained the predicted RGS2-binding sequences and of nt -850/-500 and -300/-10 that contained the HAND1-binding consensus sequence “*NNTCTG*” (HBS), and the ChIP assays were normalized with their corresponding input GAPDH. To our surprise, RGS2 unexpectedly failed to bind to any of the three *aromatase* promoter regions, whereas HAND1 bound to both regions, nt -850/-500 and nt -300/-10 (Fig. 2g), which was consistent with a previous report^27^. These data thereby reveal that RGS2 regulates *aromatase* transcription but that RGS2 does not transactivate *aromatase*.

### Transcriptional regulation of *Aromatase* by RGS2 through HAND1

**Fig. 3:**
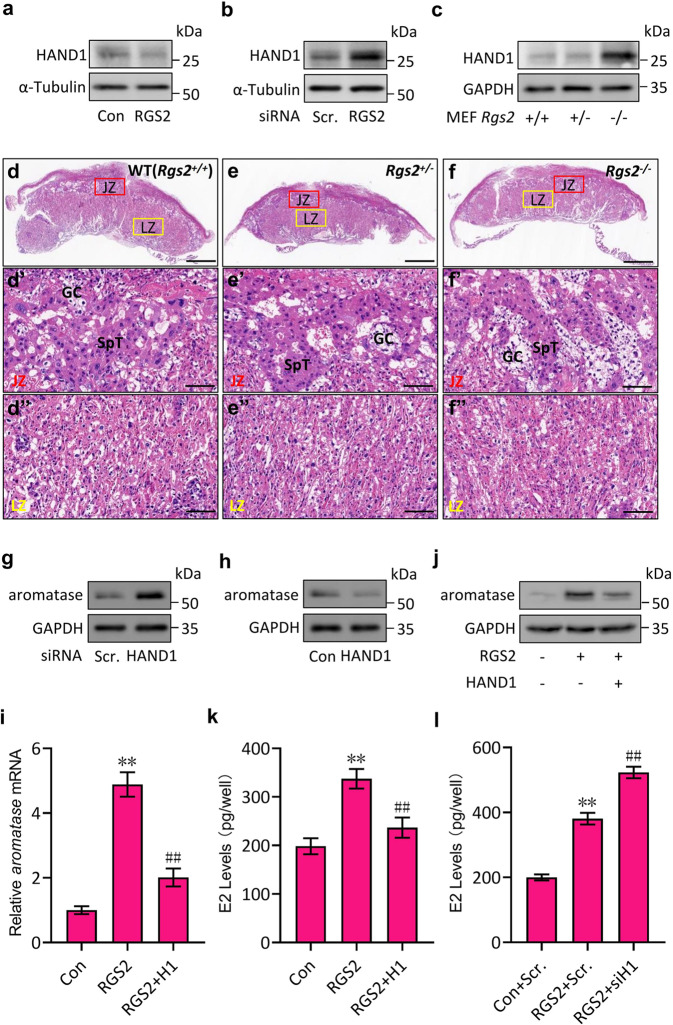
RGS2 regulates aromatase through HAND1. **a** Western blot analysis of HAND1 in JEG-3 cells transfected with Flag-RGS2 (RGS2) or control vector (Con). **b** Western blot analysis of HAND1 in JEG-3 cells transfected with RGS2 siRNA or control siRNA (Scr.). **c** Western blot analysis of HAND1 in wild-type (WT, *Rgs2*^*+/+*^), *Rgs2*^*+/−*^ and *Rgs2*^*−/−*^ MEFs at E13.5. **d**–**f** Microscopic views of *Rgs2*^*+/+*^, *Rgs2*^*+/−*^ and *Rgs2*^*−/−*^ placentas at E13.5 sectioned through their middle and by H&E staining. **d’**–**f”** Higher magnification views of the junctional zone (JZ, marked with a red square in **d**–**f**) and the labyrinth zone (LZ, marked with a yellow square in **d**–**f**) of *Rgs2*^*+/+*^, *Rgs2*^*+/−*^ and *Rgs2*^*−/−*^ placentas at E13.5 from **d**–**f**. **g** Western blot analysis of aromatase in JEG-3 cells transfected with HAND1 siRNA or control siRNA (Scr.). **h** Western blot analysis of aromatase in JEG-3 cells transfected with HAND1-Flag (HAND1) or control vector (Con). **i** Western blot analysis of aromatase in JEG-3 cells transfected with Flag-RGS2 (RGS2) alone or together with HAND1-Flag (HAND1) or control vector (Con). **j** qRT‒PCR analysis of aromatase mRNA levels in JEG-3 cells transfected with Flag-RGS2 (RGS2) alone or together with HAND1-Flag (HAND1) or control vector (Con). **k** E2 levels in the culture media of JEG-3 cells transfected with Flag-RGS2 (RGS2) alone or together with HAND1-Flag (HAND1) or control vector (Con). **l** E2 levels in the culture media of JEG-3 cells transfected with Flag-RGS2 (RGS2) or control vector (Con) together with HAND1 siRNA (siH1) or control siRNA (Scr.). RNA and protein abundance normalized to α-Tubulin or GAPDH, respectively. **p < 0.01 versus Con. or Con.+Scr., ^##^*p* < 0.01 versus RGS2 or RGS2 + Scr.; *n* = 6; error bar, SD; bars, 0.25 mm in (**d**–**f**) and 20 μm in (**d'**–**f''**). GC glycogen trophoblast cells, SpT spongiotrophoblast cells.

Next, we wanted to determine how RGS2 regulates *aromatase* transcription. To this end, we focused on HAND1, since HAND1 was shown to suppress *aromatase* transcription by directly binding to the “*NNTCTG*” sequence within its promoter region (Fig. 2g)^27^. Overexpression of RGS2 dramatically decreased the HAND1 protein levels, while knockdown of RGS2 markedly increased the protein expression of HAND1 (Fig. 3a, b). Similarly, the HAND1 protein levels were robustly increased in *Rgs2*-deficient (*Rgs2*^*−/−*^) MEFs compared with the heterozygote (*Rgs2*^*+/*−^) and wild-type (*Rgs2*^*+/+*^) cells (Fig. 3c). Intriguingly, no apparent difference was found between the *Rgs2*^*−/−*^ mouse placentas and those of littermate controls (*Rgs2*^*+/−*^ and *Rgs2*^*+/+*^) based on histological sections and H&E staining at E13.5 (Fig. 3d–f), nor did the junctional zone (JZ) or the labyrinth zone (LZ) of *Rgs2*^*−/−*^ placentas exhibit obvious abnormalities at higher magnification (Fig. 3d’–f”), indicating a different role of RGS2 in the maintenance of placental development and function between humans and mice. In contrast to the RGS2 regulation pattern of aromatase, silencing HAND1 apparently induced aromatase protein expression (Fig. 3g), while ectopic expression of HAND1 not only significantly inhibited aromatase protein expression (Fig. 3h) but also effectively attenuated RGS2-induced aromatase mRNA levels, aromatase protein levels and E2 levels (Fig. 3i–k). Consistently, knockdown of HAND1 by HAND1 siRNA (siH1) further promoted RGS2-induced E2 levels (Fig. 3l). Thus, these data reveal that RGS2 promotes *aromatase* transcription through inhibition of HAND1.

### RGS2 suppresses HAND1 expression by ubiquitination of HAND1

**Fig. 4:**
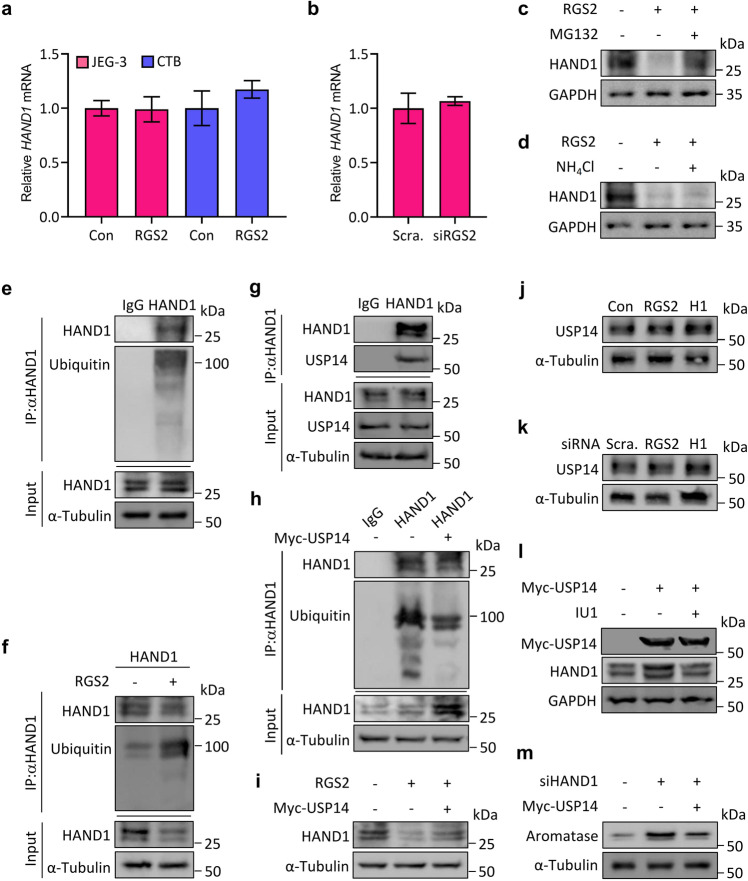
RGS2 regulates HAND1 ubiquitination. **a** qRT‒PCR analysis of the mRNA levels of *HAND1* in JEG-3 cells transfected with Flag-RGS2 (RGS2) or control vector (Con) and in human cytotrophoblasts (CTBs) infected with RGS2-expressing lentiviruses (RGS2) or control lentiviruses (Con). **b** qRT‒PCR analysis of the mRNA levels of *HAND1* in JEG-3 cells transfected with RGS2 siRNA or control siRNA (Scram.). **c** Western blot analysis of HAND1 in JEG-3 cells transfected with Flag-RGS2 (RGS2, + ) or control vector (RGS2, −) and treated with MG132. **d** Western blot analysis of HAND1 in JEG-3 cells transfected with Flag-RGS2 (RGS2, + ) or control vector (RGS2, −) and treated with NH_4_Cl. **e** Coimmunoprecipitation of endogenous HAND1 and ubiquitin in JEG-3 cells. IP: HAND1, WB: ubiquitin. **f** Coimmunoprecipitation of endogenous HAND1 and ubiquitin in JEG-3 cells transfected with Flag-RGS2 (RGS2, + ) or control vector (RGS2, −). IP: HAND1, WB: ubiquitin. **g** Coimmunoprecipitation of endogenous HAND1 and USP14 in JEG-3 cells. IP: HAND1, WB: USP14. **h** Coimmunoprecipitation of endogenous HAND1 and ubiquitin in JEG-3 cells transfected with Myc-USP14 (Myc-USP14, + ) or control vector (Myc-USP14, −). IP: HAND1, WB: ubiquitin. **i** Western blot analysis of HAND1 in JEG-3 cells transfected with Flag-RGS2 (RGS2, + ) alone or together with Myc-USP14 (RGS2, + ; Myc-USP14, + ) or control vector (RGS2, -; Myc-USP14, −). **j** Western blot analysis of USP14 in JEG-3 cells transfected with Flag-RGS2 (RGS2), HAND1-Flag (H1) or control vector (Con). **k** Western blot analysis of USP14 in JEG-3 cells transfected with RGS2 siRNA, HAND1 siRNA (siRNA H1) or control siRNA (Scra.). **l** Western blot analysis of Myc and HAND1 in JEG-3 cells transfected with Myc-USP14 (Myc-USP14, + ) or control vector (Myc-USP14, −) and treated with IU1. **m** Western blot analysis of aromatase in JEG-3 cells transfected with Myc-USP14 (Myc-USP14, + ) or control vector (Myc-USP14, −) together with HAND1 siRNA (siHAND1). RNA and protein abundance normalized to α-Tubulin or GAPDH. *n* = 6; error bar, SD.

To further clarify the mechanisms underlying HAND1 regulation by RGS2, we examined the mRNA expression of *HAND1* in response to RGS2. Unexpectedly, unlike the alterations in HAND1 protein expression, neither overexpression of RGS2 nor knockdown of RGS2 significantly affected the mRNA levels of *HAND1* in JEG-3 cells or in human cytotrophoblasts (CTBs) (Fig. 4a, b), indicating the post-translational modification of HAND1. Moreover, we found that inhibiting proteasome activity using MG132, but not lysosome activity by the inhibitor NH_4_Cl, substantially restored HAND1 protein expression in the presence of ectopic RGS2 expression (Fig. 4c, d). Thus, it is likely that HAND1 could be ubiquitinated and degraded by proteasomes in the presence of RGS2. Indeed, the results of immunoprecipitation experiments showed that endogenous HAND1 was ubiquitinated in JEG-3 cells (Fig. 4e). Therefore, these data suggest that RGS2 downregulates the protein expression of HAND1, likely through the promotion of HAND1 ubiquitination. Next, we wanted to determine whether RGS2 could directly mediate HAND1 ubiquitination. Our results revealed that overexpression of RGS2 led to enhanced polyubiquitination of HAND1 in JEG-3 cells (Fig. 4f). Intriguingly, we additionally observed that HAND1 interacted with ubiquitin-specific protease 14 (USP14), a deubiquitinating enzyme (DUB) (Fig. 4g). Thus, we postulated that RGS2 might regulate HAND1 ubiquitination through USP14. As expected, overexpression of Myc-tagged USP14 (Myc-USP14, USP14) apparently decreased HAND1 polyubiquitination and effectively reversed the inhibitory effect of RGS2 on HAND1 protein expression (Fig. 4h, i). However, neither RGS2 overexpression nor HAND1 overexpression (H1) altered USP14 protein expression (Fig. 4j, k), suggesting that RGS2 might suppress USP14 activity. This hypothesis was further demonstrated by the experiments with administration of IU1, a specific antagonist against USP14, showing that IU1 effectively attenuated the USP14-induced protein expression of HAND1 (Fig. 4l). Furthermore, overexpression of USP14 partially restored the elevated aromatase protein expression derived from HAND1 inhibition (Fig. 4m). Thus, RGS2 promotes HAND1 ubiquitination through the regulation of USP14.

### Regulation of E2 production by the RGS2-HAND1 axis in ovariectomized nude mice with JEG-3 xenografts

**Fig. 5:**
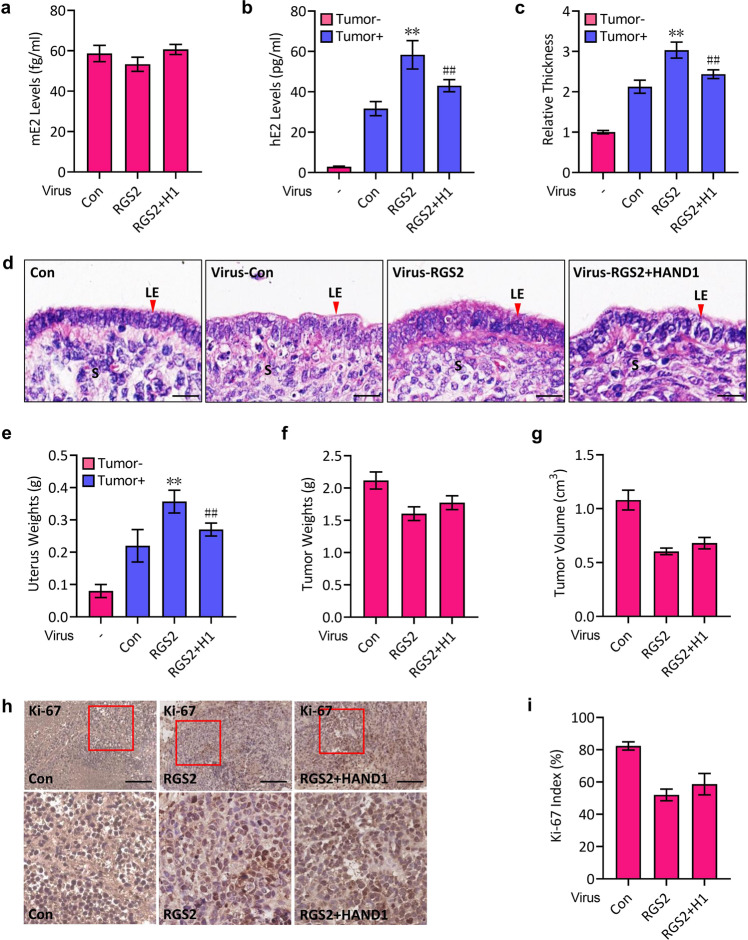
RGS2-HAND1 regulates E2 production in ovariectomized nude mice with JEG-3 xenografts. **a** Ovariectomized nude mice were subcutaneously inoculated with control lentivirus-infected (Con) or RGS2-expressing lentivirus-infected (RGS2) or RGS2 and HAND1-expressing lentivirus-infected (RGS2 + H1) JEG-3 cells. Mouse E2 (mE2) levels in circulation were examined 14 days after inoculation. **b** Human E2 (hE2) levels in circulation were examined in nude mice with the same treatments as in **a** and in ovariectomized nude mice without JEG-3 xenografts. **c**, **d** H&E staining of uteri from ovariectomized nude mice in **b**. **c** Statistical analysis of uterus epithelial thickness in **d** (relative to ovariectomized nude mice without xenografts). LE luminal epithelium, S stroma. **e** Uterine weights in ovariectomized nude mice of **b**. **f**, **g** Tumor weights (**f**) and tumor volume (**g**) in ovariectomized nude mice of **b**. **h** Immunohistochemistry staining for Ki-67 of tumor cells from control xenografts (Con) or RGS2-expressing xenografts (RGS2) or RGS2 and HAND1-expressing xenografts (RGS2 + HAND1). **i** Quantification and statistical analysis of the Ki-67 index in **h**. **p < 0.01 versus Con, ^##^*p* < 0.01 versus RGS2, *p < 0.05 versus Con; *n* = 6; error bar, SD; bars, 10 μm in **d** and 100 μm in **h**.

To additionally test the functional relevance of the RGS2-HAND1-aromatase signaling axis in E2 regulation by trophoblasts, we generated xenografts derived from JEG-3 cells that were infected with RGS2-expressing lentiviruses and/or HAND1-expressing lentiviruses in ovariectomized nude mice and monitored the uterine responses. Although either overexpression of RGS2 or overexpression of RGS2 together with HAND1 did not cause significant morphological changes in the uterus of ovariectomized nude mice without JEG-3 xenografts (data not shown) and the ovariectomy resulted in trace serum levels of murine E2 (mE2, Fig. 5a), the existence of JEG-3 cell-derived xenografts in the ovariectomized mice produced abundant human E2 (hE2) in the serum, whose levels were comparable to the physiological serum concentrations in mice, leading to the thickened uterine epithelial layer and the increased uterus weights (Fig. 5b–e). In addition, the RGS2-overexpressing JEG-3 xenografts in the ovariectomized nude mice (Virus-RGS2) produced more human E2 (hE2) in the mouse serum, caused a thicker epithelial layer and increased the uterus weights compared with those with control xenografts (Virus-Con) (Fig. 5b–e). However, overexpression of RGS2 together with HAND1 (Virus-RGS2 + H1 group in the figure) significantly restored the levels of hE2 derived from RGS2 alone-overexpressing JEG-3 xenografts (Virus-RGS2) and reduced the RGS2-enhanced thickness of the uterine epithelial layer and uterus weights (Fig. 5b–e), although it did not affect the weights and volumes of the xenografts (Fig. 5f, g), nor did it change the proliferative status of the xenograft cells (Fig. 5h, i). Of note, overexpression of RGS2 suppressed tumor weights, tumor size and tumor cell proliferation to some extent compared with those of the control group (Virus-Con) (Fig. 5f–i), suggesting a suppressive role of RGS2 in tumorigenesis, which was consistent with a previous report showing that RGS2 inhibits tumor cell growth^30^. In summary, HAND1 reduces the production of E2 by RGS2-overexpressing JEG-3-derived xenografts in mice, resulting in a reduced uterine response.

### Modulation of RGS2 activity by aromatase

**Fig. 6:**
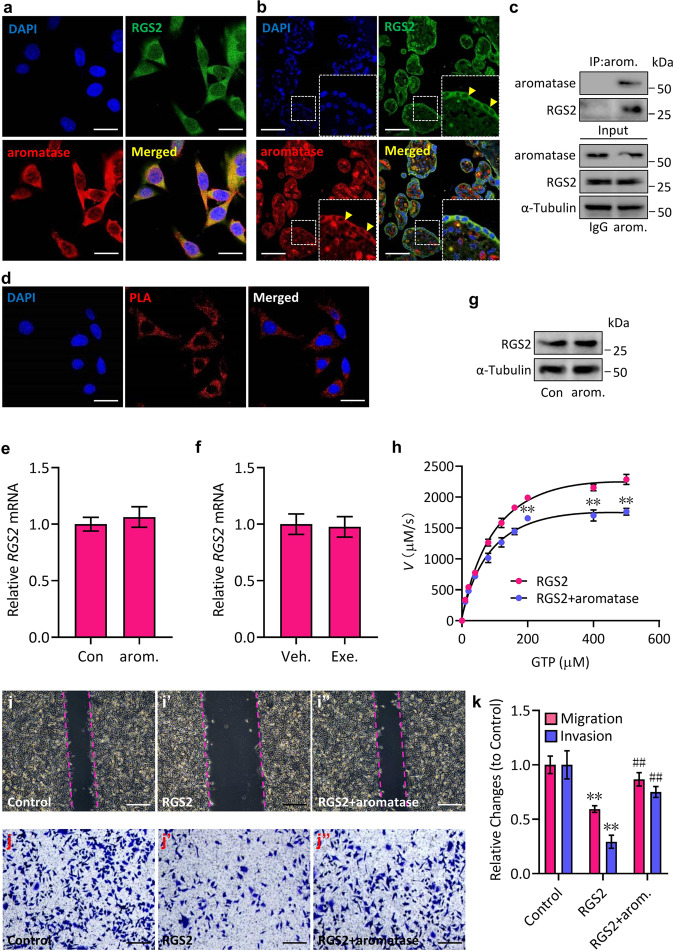
Aromatase inhibits RGS2 activity. **a** Immunofluorescence staining of the RGS2-derived signal (green) and aromatase-derived signal (red) in JEG-3 cells. Nuclei were stained with DAPI. **b** Immunofluorescence staining of the RGS2-derived signal (green) and aromatase-derived signal (red) in human placental tissues. Nuclei were stained with DAPI. **c** Coimmunoprecipitation of endogenous aromatase and RGS2 in JEG-3 cells. IP: aromatase (arom.), WB: RGS2. **d** Proximity ligation assay (PLA) in human placental tissues. Red spots indicate the RGS2-aromatase protein interaction. Nuclei were stained with DAPI. **e** qRT‒PCR analysis of the mRNA levels of *RGS2* in JEG-3 cells transfected with aromatase (arom.) or control vector (Con). **e** qRT‒PCR analysis of the mRNA levels of *RGS2* in JEG-3 cells transfected with aromatase (arom.) or control vector (Con). **f** qRT‒PCR analysis of the mRNA levels of *RGS2* in JEG-3 cells treated with exemestane (Exe.) or vehicle (Veh.). **g** Western blot analysis of RGS2 in JEG-3 cells transfected with aromatase (arom.) or control vector (Con). **h** In vitro GTP hydrolysis by RGS2 or by RGS2 and aromatase. Purified RGS2 and aromatase proteins were incubated with increasing amounts of GTP at 37 °C for 3 h. **i**–**i''** Wound healing assay of JEG-3 cells transfected with RGS2 or control vector in combination with aromatase. **j**–**j''** Matrigel invasion assay of JEG-3 cells transfected with RGS2 or control vector in combination with aromatase. **k** Quantitative analysis of **i**–**j''**. RNA and protein abundance normalized to that of α-Tubulin. **p < 0.01 versus Gαi or control, ^##^*p* < 0.01 versus Gαi + RGS2 or RGS2, *n* = 6; error bar, SD; bars, 60 μm in (**a**, **d**), 10 μm in (**b**), 15 μm in (**i**–**i''**) and 60 μm in (**j**–**j''**).

To determine whether aromatase reciprocally mediates RGS2 expression, we subsequently observed the protein localization of RGS2 and aromatase in trophoblasts and tested the possible protein-protein interaction between RGS2 and aromatase. Immunofluorescence staining data revealed that both RGS2 and aromatase were mainly present in the cellular cytoplasm and that RGS2 was colocalized with aromatase in both JEG-3 cells and the trophoblast of human term placental tissues (Fig. 6a, b), which was further examined by coimmunoprecipitation experiments using JEG-3 cells in vitro and was also verified by a proximity ligation assay (PLA) using human placental tissues in vivo (Fig. 6c, d), demonstrating that RGS2 bound with aromatase. However, unexpectedly, either aromatase overexpression or administration of exemestane (Exe.), an antagonist against aromatase, failed to regulate the mRNA and protein expression of aromatase (Fig. 6e–g, data not shown), which was consistent with the results of E2 and RGS2 expression (Fig. 1q, r), proving that aromatase did not regulate RGS2 expression. Given that RGS2 acts as a multifunctional RGS protein that regulates multiple G-protein linked signaling pathways *via* its RGS domain, which possesses GTPase activating protein (GAP) activity for members of the Gi (Giα1-3, Goα, Gzα, Gtα and Ggustα) and Gq (Gqα, G11α, G14α and G16α) subfamilies^31^, we next tested the effect of aromatase on RGS2 GAP activity by performing in vitro GTP hydrolysis assays with the determination of the Michaelis‒Menten kinetic properties of purified RGS2 and aromatase. Our results showed that RGS2 displayed GTPase activity in a GTP dose-dependent manner (Fig. 6h), whereas the presence of aromatase effectively abolished the catalytic efficiency of RGS2 (Fig. 6h), suggesting that aromatase repressed RGS2 activity. As expected, overexpression of RGS2 significantly inhibited JEG-3 cell migration and invasion, which was consistent with previous reports^32,33^, while coexpression of aromatase effectively attenuated the RGS2-suppressed migratory and invasive capacities of JEG-3 cells (Fig. 6i–k). Thus, aromatase negatively regulates RGS2 GAP activity.

### Aromatase expression correlates with RGS2 levels in PE

**Fig. 7:**
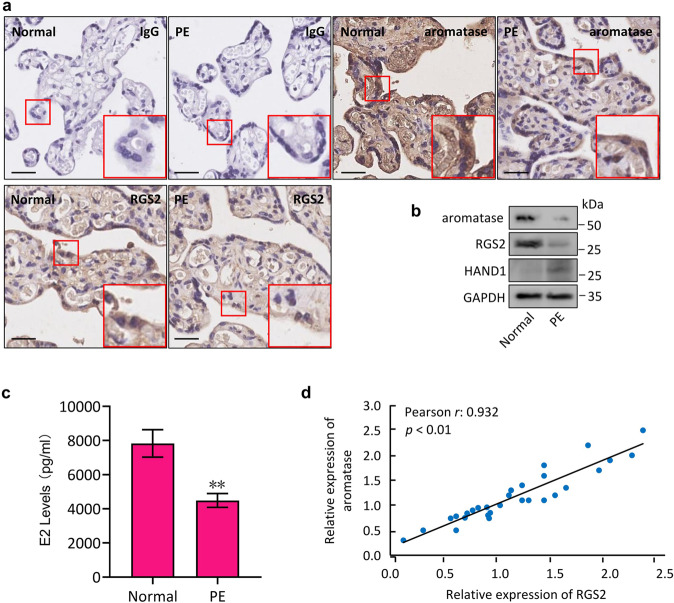
Aromatase expression correlates with RGS2 levels in PE. **a** Immunohistochemistry staining for aromatase and RGS2 in placental samples from patients with PE and from normal pregnancies (Normal). IgG was used as a negative control. **b** Western blot analysis for aromatase, RGS2 and HAND1 in placental samples from patients with PE and from normal pregnancies (Normal). **c** E2 levels in blood samples from patients with PE and from normal pregnancies (Normal). **d** The correlation between RGS2 and aromatase in placentas was determined by Pearson’s correlation coefficient (r: 0.932, *p* < 0.01). Protein abundance was normalized to GAPDH. **p < 0.01 versus normal; *n* = 6 (**a**–**c**); error bar, SD; bars, 100 μm.

To further elucidate the relationship between RGS2 and aromatase in PE, we detected RGS2 expression and aromatase expression in 15 paired placentas from patients with PE and women with normal pregnancies. Immunohistochemistry staining showed that the levels of RGS2 were apparently lower in PE than in normal pregnancies (Fig. 7a), and similarly, aromatase levels were obviously decreased in the PE group, which could be additionally verified by Western blots, showing decreased protein expression of RGS2 and aromatase but increased HAND1 protein expression in preeclamptic tissues (Fig. 7b). Likewise, the E2 levels in women with PE were significantly lower than those in women with normal pregnancies (Fig. 7c). More importantly, the protein expression of RGS2 in the 30 placentas positively correlated with aromatase levels (Pearson r: 0.932, *p* < 0.01, Fig. 7d). These data finally indicated that RGS2 regulates aromatase expression and that their correlation could be determined in clinical samples.

## Discussion

The decreased levels of RGS2 have been shown to be associated with PE, but how the downregulated RGS2 expression affects placentation and contributes to the progression of diseases such as PE remains largely unknown. In mouse models, the reduced mRNA levels of *Rgs2* are sufficient to cause features of the disorder, and mutations that decrease vascular Rgs2 expression may be a predisposition to lowered uterine blood flow^16,23^. In our present study, we demonstrate that the reduced levels of RGS2 cause the upregulation of HAND1 protein expression that leads to the inhibition of aromatase and thereby results in decreased E2 production, which may give rise to the development of PE and failure in pregnancy maintenance.

PE, in particular, is one of the most feared complications of pregnancy. While the cause of PE is still debated, clinical and pathological studies suggest that the placenta is central to the pathogenesis of this syndrome^34,35^. Comparison of the gene expression between normal placenta and preeclamptic placenta may provide some clues for the discovery of candidate genes that are associated with PE. In our present study, we have proven that the expression of RGS2 and aromatase is decreased while HAND1 levels are increased in preeclamptic tissues by using both in vitro experiments and human clinical placental samples. However, by a xenograft model in nude mice, we additionally provide potential evidence that the decreased E2 levels by reduced RGS2 are at least partly due to the upregulation of HAND1, which suppresses aromatase expression and is a rate-limiting enzyme in E2 biosynthesis from androgen by trophoblasts. Nevertheless, it is uncertain whether interruption of the RGS2-HAND1-aromatase signaling axis gives rise to PE, which is worthy of further investigation.

*Hand1*, encoding a basic helix-loop-helix (bHLH) transcription factor, is involved in the regulation of the determination and differentiation of several cell lineages during placentation in mice^36^. *Hand1* deficiency in mice caused developmental arrest at E7.5 due primarily to placental defects that include a block in trophoblast giant cell differentiation and a smaller ectoplacental cone^37,38^, suggesting the essential role of HAND1 during placentation and embryogenesis. Although there are obvious differences between the human placenta and mouse placenta in placental morphogenesis and functions^39^, our recent study revealed that HAND1 also functions in the human placenta, particularly in the regulation of steroidogenesis from cholesterol^27^. In the present study, we discovered an upstream regulator of HAND1, RGS2, which promotes HAND1 protein stability by inhibiting ubiquitin-mediated degradation through regulation of USP14. These findings also imply that, in addition to its role in the murine placenta, HAND1 is required for the appropriate regulation of human trophoblast functions.

Deubiquitinating enzymes (DUBs) act antagonistically to protein ubiquitination and play a key role in regulating ubiquitin signaling events such as protein stability and localization^40^. USP14, a major regulator of the proteasome and one of three proteasome-associated deubiquitinating enzymes, plays an active role in the recycling of ubiquitin from proteins targeted to proteasomes^41^. Previous studies have revealed that the reduced levels of Usp14 in mice are retarded for growth and present a series of behavioral disorders, including resting tremor and hind-limb paralysis, and loss of *Usp14* (*Usp14*^*−/*−^) results in embryonic death at E13.5 owing to the failure in development, particularly various defects in neuronal development^42,43^, suggesting the requirement for USP14 in the maintenance of the functions of the neural system. Given that USP14 is widespread within different tissues^44–47^, USP14 may be essential for the proper functioning of various cellular events. In the present study, we have provided evidence that USP14 is involved in human placental functions, which fulfills the diverse role of USP14 during development. However, the specific regulatory sites in HAND1 that are recognized and affected by USP14 remain unknown and merit further investigation.

As an estrogen synthetase, aromatase produces 18-carbon estrogens from 19-carbon androgens. Aromatase is well known for its roles in reproduction and reproductive system diseases, and its deregulation leads to abnormal E2 levels that give rise to malignancies and diseases of the breast, ovary and endometrium^48^. During pregnancy, aromatase-mediated E2 production is required for placental development and fetal growth. At the end of pregnancy, maternal blood concentrations of E2 are almost 100-fold higher than those of nonpregnant women, reaching 30 nmol/L^49,50^, whereas placental aromatase deficiency can be detected in women with PE at delivery, and decreased levels of E2 are considered to be closely associated with PE^51,52^, suggesting the essential role of aromatase in pregnancy. In our present work, we identified the possible mechanisms by which aromatase is suppressed and confirmed the correlations between RGS2 and aromatase in both normal placental tissues and preeclamptic tissues.

Overall, using a human JEG-3 cell model and a xenograft model as well as clinical samples, we found that RGS2 plays an important role in human placentation and pregnancy maintenance. The decreased expression of RGS2 is associated with PE development and results in the lowered levels of E2 through USP14-mediated upregulation of HAND1 and subsequent suppression of aromatase by HAND1, while the downregulated aromatase relieves its inhibition of RGS2 activity. Thus, the RGS2-aromatase loop acts as a critical event in supporting the development and physiological function of the human placenta.
